# SOFTPEERS: feasibility, acceptability, and preliminary effectiveness of a peer-led intervention to prevent binge drinking in adolescents. Results from a mixed-methods pilot cluster-randomized controlled trial

**DOI:** 10.3389/fpubh.2026.1791492

**Published:** 2026-07-08

**Authors:** Florence Cousson-Gélie, Olivier Lareyre, Emilie Charton, Marie Cholley-Gomez, Sarah Ricupéro, Florian Jeanleboeuf, Marie Boulvin, Adrien Minotte, Delphine Poquet, Amélie Anota, Agnès Dumas, Marion Carayol, Mathieu Gourlan

**Affiliations:** 1 Epsylon Laboratory EA4556, Université de Montpellier Paul-Valéry, Montpellier, France; 2 Epidaure Prevention Department, Montpellier Cancer Institute, Montpellier, France; 3 Department of Human and Social Sciences, Centre Léon Bérard, Lyon, France; 4 INSERM, Aix Marseille Univ, IRD, ISSPAM, SESSTIM (Economic and Social Sciences of Health and Medical Information Processing), CALIPSO Team, Marseille, France; 5 Direction of Clinical Research and Innovation, Centre Léon Bérard, Lyon, France; 6 Youth – Physical and Sports Activity – Health Laboratory (JAP2S), University of Toulon, Toulon, France

**Keywords:** acceptability and adoption, adolescents, binge drinking, cluster randomized controlled trial, feasability, mixed-methods school-based intervention

## Abstract

**Introduction:**

Population surveys indicate that binge drinking is highly prevalent among adolescents worldwide and is associated with a wide range of harmful consequences. School-based peer-led interventions are considered a promising strategy, as adolescents are especially receptive to preventive messages delivered by their peers. The present pilot study aimed to assess the feasibility, acceptability, and preliminary effectiveness of the SOFTPEERS program, a peer-led intervention designed to prevent binge drinking in French high school students.

**Methods:**

A mixed-methods design was used. The quantitative component consisted of a cluster-randomized controlled trial involving 2,021 students in grades 10–11 (15–18 years) from eight high schools (four intervention, four control) assessing the 6-month impact of the SOFTPEERS program on binge drinking prevalence. The qualitative component consisted of a process evaluation conducted through 13 individual interviews and 8 focus groups, examining delivery conditions, perceptions of individuals involved, and contextual factors influencing implementation. Quantitative data were analyzed using generalized linear mixed models with a logit link function, and qualitative data through thematic analysis.

**Results:**

Quantitative analyses revealed a significant time x group interaction on binge drinking prevalence (*b* = −0.08, 95% confidence interval [−0.12, −0.04]), indicating that the increase in binge drinking prevalence in the past 30 days between baseline and 6-month follow-up was significantly smaller in the intervention group (+1.3 percentage points) than in the control group (+9.2 percentage points). Qualitative analyses indicated that peer educators considered the program appropriate, enjoyable, and personally enriching. Peer recipients appreciated the peer prevention approach, considering it more open and pleasant than an adult-led approach, and demonstrating a certain degree of effectiveness in terms of knowledge gained and behavioral regulation. Members of the high school staff also appreciated the peer-to-peer approach. However, logistical and scheduling constraints required local adaptations from both professional health educators and peer educators to ensure program delivery.

**Discussion:**

These findings suggest that the SOFTPEERS program is a feasible, acceptable, and potentially effective peer-led intervention to prevent binge drinking among French high school students. In light of these results, a full-scale cluster-randomized controlled trial should be conducted to confirm the program’s effectiveness.

**Clinical trial registration:**

https://doi.org/10.1186/ISRCTN11897676, ISRCTN11897676.

## 1 Introduction

Alcohol use is one of the most frequent causes of preventable death worldwide, accounting for approximately 2.6 million deaths in 2019 (1). Regular alcohol use typically begins during adolescence, with more than a quarter (26.5%) of adolescents aged 15–19 years considered current drinkers, representing approximately 155 million individuals worldwide (1). Binge drinking is one of the most common risky behaviors among adolescents. Binge drinking is defined as the consumption of at least 60 grams of pure alcohol (about five alcoholic drinks) on a single occasion (1). This drinking pattern is associated with numerous negative outcomes at both individual and interpersonal levels, including blackouts, illicit drug use, interpersonal violence, risky sexual behaviors, and poor academic performance (2, 3). Additionally, binge drinking during adolescence is also linked to long-term consequences, such as an increased risk of developing substance use disorders in adulthood (4). Yet, in many high-income countries (e.g., Australia, Canada, New Zealand, and the United States), more than 17% of adolescents aged 15–19 years reported having engaged in at least one binge drinking episode in the past 30 days (1). In the European region, binge drinking rates among adolescents are even higher (1). For instance, in France, 36.6% of 17-year-olds reported engaging in at least one binge drinking episode, and nearly 60% reported being drunk in the past 30 days (5). In this context, developing and evaluating interventions to prevent binge drinking among adolescents is considered a public health priority (6).

Recent systematic reviews and meta-analyses have reported a significant, yet modest, impact of seven parent-based and 16 theory-based interventions on adolescents’ binge drinking (7, 8). However, none of these reviews identified interventions based on a peer-led approach within school settings, which has been proposed as a potentially effective strategy to address substance use among adolescents (9). The peer-led approach refers to a strategy whereby peers, rather than adults or professionals, act as the agents who relay information or skills to other members of their group (9, 10). When operationalized into a structured peer-led intervention, this typically involves selecting and training some group members as peer educators, who then deliver the intervention to their peers, referred to as peer recipients (9). The relevance of relying on peers is rooted in adolescence, a period when peer relationships become central to development, with increased sensitivity to peer approval and stronger sensitivity for peer opinions (11). While peer influence can promote binge drinking in adolescents—for instance, peer alcohol use is one of the strongest correlates of adolescent drinking behavior (12, 13)—it can also serve as a protective factor through peer education (10). Peers are often perceived as credible sources of support due to their shared cultural background, empathetic understanding of experiences of their counterparts, and their ability to act as role models in promoting healthy behaviors (14). Adolescents are also more likely to seek help and advice from informal sources such as peers rather than adults, which may enhance the acceptability and impact of prevention messages delivered through peer-led approaches (9). In addition, the school environment offers a particularly relevant context for peer-led interventions, as it brings together daily peer interactions and social dynamics within a wide and diverse audience of students, including those from socially and economically disadvantaged backgrounds (9, 15). Beyond peer interactions, the involvement of educational staff, such as teachers, may serve as a valuable complement to peer-led approaches in school-based interventions. Rather than being seen as operating in distinct, unrelated and even opposite spheres (16), teachers and peer educators can be understood as playing mutually reinforcing roles within a peer-led intervention (17). While peer educators are central to delivering the intervention among peer recipients, teachers can act as institutional enablers, for instance by helping to legitimize the peer-led intervention and foster a supportive environment within the school (18).

A meta-analysis found that six interventions relying upon a peer-led approach with adolescents were associated with a 20% decrease in the odds of alcohol use (19). However, the specific impact of peer-led interventions on preventing binge drinking remains largely unknown. Binge drinking represents a distinct pattern of alcohol consumption, which also calls for considering specific contextual factors that may be associated with its occurrence (20). From this, peer-led interventions targeting adolescent binge drinking may face specific challenges in their implementation and potential impact. From a behavioral perspective, binge drinking may present certain characteristics that at least partially distinguish it from general alcohol use. For instance, binge drinking in adolescence often takes place in situations that create risk-conducive opportunities, such as parties or group gatherings, where adolescents may engage in binge drinking even in the absence of prior intention to do so (21). The spontaneous nature of binge drinking may notably challenge peer leaders in designing interventions that allow peer recipients to mentally project themselves into such situations, and may also limit the ability of peer recipients to act in line with the insights or intentions formed during the intervention. Otherwise, binge drinking is also characterized by particularly strong ritualized social and societal dimensions, often framed as group challenges or rites of passage that contribute to adolescents’ social integration within peer groups, communities and the broader youth culture (22). This cultural dimension may be particularly salient in France, where alcohol, and especially wine, holds a highly valued place in national identity, cultural heritage, and economic activity (23), creating a cultural environment in which alcohol use is widely accepted and binge drinking among French adolescents is normalized and widespread (5, 13). In addition, because binge drinking is a socially valued and normalized behavior among adolescents (22, 23), some may be reluctant to engage with an intervention targeting this behavior (24), and it remains unclear whether a peer-led format would be sufficient to overcome this reluctance. Finally, beyond the public health relevance of addressing adolescent binge drinking (6), it is also important to consider whether high school decision-makers (e.g., high school headmasters) are willing to prioritize this issue and support the implementation of peer-led interventions within school settings. Moreover, limited evidence is available regarding whether educational staff, such as teachers, are willing or able to become involved during the early stages of a peer-led intervention addressing adolescent binge drinking, particularly in introducing the program to students and fostering their initial engagement and reflection (17). Taken together, these elements underscore the importance of conducting a pilot study to assess whether a peer-led intervention can be effectively implemented within high schools, identify key obstacles and facilitators to its implementation, and evaluate its potential impact on adolescent binge drinking.

The present pilot study aimed to assess the feasibility, acceptability, and preliminary effectiveness of the SOFTPEERS program, a peer-led intervention designed to prevent binge drinking in French high school students. Following existing guidelines (25), the present pilot study focused on five key areas of investigation. These included:

1. Demand: To what extent are high schools and students (i.e., peer educators and recipients) willing to engage with the SOFTPEERS program?
2. Acceptability: How is the SOFTPEERS program perceived in terms of relevance, appeal, and perceived value by students (i.e., peer educators and recipients) and program deliverers (i.e., professional health educators, project team members, and high school staff)?
3. Implementation: To what extent can the SOFTPEERS program be delivered as intended within the organizational and logistical constraints of real-world high school settings?
4. Adaptation: To what extent was the SOFTPEERS program adapted to local constraints, practices and contexts of participating high schools?
5. Preliminary effectiveness: Does the SOFTPEERS program show preliminary effectiveness in preventing binge drinking among students (i.e., peer educators and recipients) over a 6-month period?

## 2 Materials and methods

### 2.1 Recruitment

Between June and September 2018, the SOFTPEERS study was proposed to 57 high schools in the five departments of the Montpellier Academy (Aude, Gard, Hérault, Pyrénées-Orientales, and Lozère), in the south of France, that met the following predefined criteria: public status, general or technological curriculum, and a minimum enrollment of 200 students across 10th and 11th grades. Heads of high schools (i.e., school headmasters or vice headmasters) were able to contact the research team for further information about the study. Ultimately, nine high schools agreed to participate in the study. In each participating high school, a steering group was established to facilitate the implementation of the SOFTPEERS program. This group generally included the school headmaster or vice headmaster, the principal education advisor, and the school nurse.

**Figure 1:**
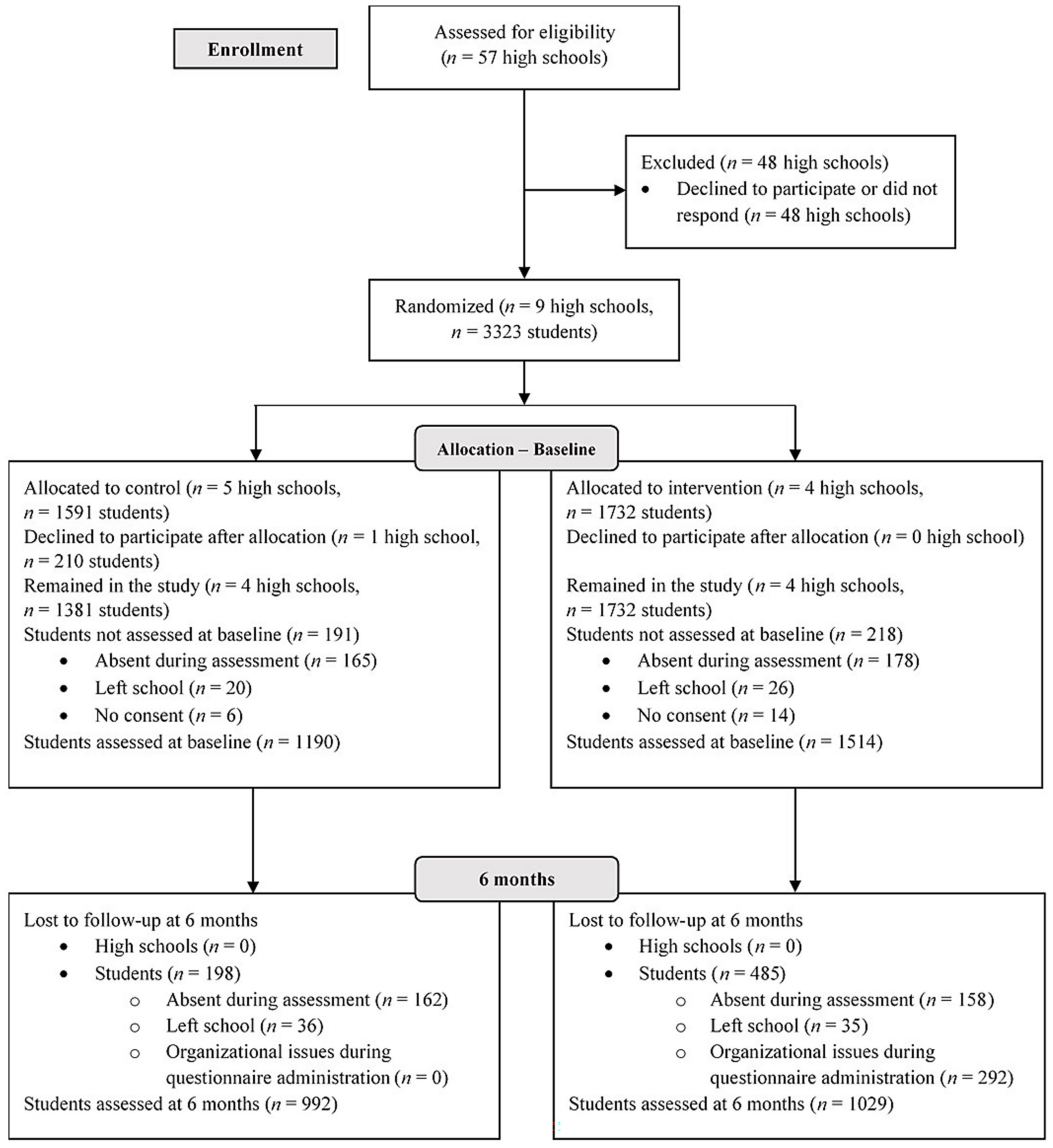
Flow diagram of participants throughout the SOFTPEERS study. Baseline, pre-intervention; 6-month follow-up, post-intervention.

Before randomization, these nine high schools were stratified according to their level of social deprivation using the French version of the European Deprivation Index (F-EDI), a validated ecological indicator of area-level deprivation in France (26). This stratification was conducted as socio-economic factors have been shown to be associated with adolescents’ alcohol use (5), binge drinking (27), and engagement with school-based prevention programs (28). The F-EDI combines individual-level indicators of material deprivation (e.g., difficulty paying bills, inability to afford meat or fish every other day) with census-based variables measured at the neighborhood level (e.g., unemployment rate, proportion of residents with low educational attainment) (26). These data are aggregated at the IRIS level (i.e., Ilots Regroupés pour l’Information Statistique; the smallest French administrative unit for census data), allowing the calculation of a deprivation score for each geographic area (26, 29). The F-EDI score of each high school was categorized into quintiles, ranging from Q1 (lowest deprivation) to Q5 (highest deprivation) (26). To ensure allocation concealment, an independent statistician, not involved in the recruitment of high schools or students, generated the random allocation sequence with high schools as the unit of randomization. High schools were randomized in a 1:1 ratio within strata defined by their level of social deprivation: low (F-EDI ≤ 3) or high (F-EDI ≥ 4) (*n* intervention = 4, *n* control = 5) (see Figure 1).

### 2.2 Interventions

#### 2.2.1 The SOFTPEERS program

In high schools assigned to the intervention group, the SOFTPEERS program, a peer-led intervention designed to prevent binge drinking, was implemented during the 2018–2019 school year in two successive phases. During the first phase (December 2018–January 2019), volunteer teachers or educational staff members (e.g., homeroom teachers, biology teachers, or school supervisors) delivered a 50-min session in each 10th- and 11th- grade class. Designed to stimulate open discussion about alcohol and support personal reflection, this session also served to introduce the SOFTPEERS program and identify volunteer peer educators among 10th- and 11th-grade students. In this session, teachers were intentionally given a facilitating rather than a lecture-based role, consistent with evidence supporting interactive teaching approaches when involving teachers in adolescent alcohol prevention programs (30, 31). Of note, participating teachers received a short instructional guide developed by the research team, which included session objectives, an overview of the question-and-answer slideshow used to structure the class discussion, and tips for encouraging student reflection (see below). In the second phase (February–May 2019), the recruited peer educators were supported by a professional health educator through six 50-min guidance sessions to design and implement binge drinking prevention actions for their peers within their high school. In line with some previous peer-led interventions (32), the content of these guidance sessions was informed by the United Nations Office on Drugs and Crime recommendations for peer-led interventions in adolescents (33), structured around five key principles: (i) being culturally appropriate (e.g., drawing on the social contexts and occasions in which adolescents typically encounter alcohol, such as high school parties or local festive events); (ii) being developmentally appropriate (e.g., designing prevention messages and formats suited to the interests and communication styles of high school students); (iii) providing accurate information (e.g., relying on factual content about the effects and risks of binge drinking rather than moralizing or fear-based messages); (iv) being based on experiential learning (e.g., encouraging peer educators’ own creativity and initiative when designing their prevention action); and (v) relying on staff professionalism (e.g., the structuring and supervision of the guidance sessions by a professional health educator) (33). Also, in line with the support provided to teachers, all professional health educators involved participated in a one-day training session prior to the implementation of the SOFTPEERS program (i.e., November 2018), and received a methodological guide developed by the research team. The guide included detailed outlines for each of the six guidance sessions, including the specific objectives and suggested activities, practical advice for supporting group dynamics, and ready-to-use support materials. While providing a clear structure, the guide also encouraged educators to adapt the sessions to the dynamics and level of engagement of the peer educator group (e.g., initiating a round-table to promote more balanced participation when some students are less inclined to speak).

During the first phase, the 50-min session led by the teacher consisted of two main activities. The first activity was a 30-min open discussion session, supported by a question-and-answer slideshow. The objective was to initiate a collective discussion on alcohol, allowing students to express their existing knowledge and beliefs, while also receiving factual information on physiological effects (e.g., how alcohol is processed by the liver, the time required to eliminate a standard drink) and risks associated with alcohol consumption (e.g., risk of dependence, impaired judgment) (34). The activity also addressed common sources of influence, such as French cultural traditions, alcohol marketing, and discounted pricing (e.g., happy hours) (34). The second activity consisted of a 20-min two-step reflective exercise. First, each student was invited to complete a short worksheet designed to encourage personal introspection regarding their own alcohol use and their perceived freedom to choose whether or not to consume (35). This individual reflection remained private and was not collected or reviewed by the teacher. In a second step, a short class discussion was held around the question: “What advice would you give to someone who wants to avoid binge drinking?” Students were encouraged to share practical strategies and recall key messages from the previous slideshow. Throughout the session, the teacher acted as a facilitator rather than a lecturer, encouraging students to participate freely, validating diverse points of view, and fostering an atmosphere of respect and openness. Discussions were guided using an informational tone, avoiding judgment or pressure, and highlighting the underlying mechanisms behind alcohol marketing and social norms. The goal was to help students develop critical thinking and personal reflection, regardless of their current behaviors or experiences with alcohol. Lastly, at the end of the session, students were also briefly informed about the possibility of becoming peer educators as part of the SOFTPEERS program. The teacher highlighted that some of the ideas shared during the collective discussion could be further developed through peer-led prevention actions. Students interested in participating were invited to contact the teacher in the following days and register voluntarily.

At the beginning of the second phase (February–March 2019), the peer educators recruited during the first phase in each high school participated in six 50-min guidance sessions, supervised by a professional health educator, to design a binge drinking prevention action for peer recipients. Group size ranged from three to seven students, depending on the high school. In sessions 1 and 2, peer educators reflected on their own representations of alcohol, identified and discussed key factors influencing binge drinking among adolescents (e.g., peer pressure, alcohol marketing), and were introduced to the principles of peer education. They also began selecting prevention messages they found relevant to share with their peer recipients. When appropriate, the health educator could encourage students to build upon the reflections and discussions initiated during the initial session led by a teacher in the first phase of the intervention. In session 3, peer educators explored decision-making in social situations. Through practical scenarios, they practiced how to assertively refuse alcohol and how to support their peers in resisting social pressure. This session was designed to strengthen their confidence and sense of legitimacy in adopting a peer educator role focused on binge drinking prevention. In session 4, peer educators outlined their prevention project by choosing a central message and a dissemination format (e.g., posters, videos, or short scenes acted out by students). They also clarified their objectives and specified how they intended to deliver their message within the school (e.g., in individual classrooms, in shared spaces). In sessions 5 and 6, peer educators worked collaboratively on the production of their prevention tools, finalized the materials, and rehearsed how they would deliver their actions. Session 6 also included relaxation exercises to help them prepare for the presentation phase. Throughout the six sessions, the professional health educators supporting their respective peer educator groups played a facilitating role by providing structure (e.g., organizing activities and managing time), fostering group cohesion, and guiding students in the design and implementation of their prevention action. The health educators also fostered peer educators’ autonomy, creativity, and critical thinking without prescribing content or solutions. When necessary, they helped them refine their ideas to ensure alignment with the core objective of the program (i.e., preventing binge drinking). At the end of the second phase (April–May 2019), peer educators set up their binge drinking prevention action for peer recipients within their high school. Each group was free to choose the dissemination format (e.g., posters, videos, or short scenes acted out by students) and setting of their action (e.g., in classrooms, shared spaces) depending on the project developed during the guidance sessions (see above). The length and organization of the prevention actions varied: some were punctual and centered on live interaction (e.g., a video followed by a debate, interactive stands), while others were more extended over time (e.g., posters displayed in shared spaces for several weeks). Regardless of the chosen approach, peer educators were encouraged to create opportunities for dialog with peer recipients, for instance by first presenting and discussing a poster in classrooms before displaying it in a shared space of the high school. Peer educators were encouraged to take initiative, while professional health educators remained available to provide support if needed.

#### 2.2.2 Passive control group

During the protocol, students in the control group did not receive any alcohol-related intervention. Staff members from high schools assigned to the control group were invited to implement the SOFTPEERS program at the beginning of the school year following the end of the protocol (i.e., the 2019–2020 school year).

### 2.3 Study design and data collection

#### 2.3.1 Design

This pilot study used a mixed-methods approach to evaluate the feasibility, acceptability, and preliminary effectiveness of the SOFTPEERS program, a peer-led intervention to prevent binge drinking among French high school students. The quantitative component consisted of a cluster-randomized controlled trial, with randomization performed at the school level, to assess the preliminary effectiveness of the program. The qualitative component consisted of a process evaluation conducted in the intervention group, to examine the feasibility, acceptability, and implementation of the program. All students enrolled in 10th and 11th grades (aged 15–18) in the participating high schools were eligible to participate in the study. Exclusion criteria were as follows: refusal to participate; inability to read or speak French; or absence at the baseline assessment. Of note, some students were involved in both qualitative (i.e., focus groups) and quantitative (i.e., questionnaires) data collection. Parental consent and student assent were obtained prior to the beginning of the study.

The overall study protocol was conducted between November 2018 and November 2019, and included SOFTPEERS program delivery, quantitative data collection at baseline and follow-up, and qualitative data collection. The study was approved by the Institutional Review Board of the French Institute of medical research (IRB00003888, IORG0003254, FWA00005831).

#### 2.3.2 Quantitative data collection

Quantitative data collection consisted of comparing the intervention group to a passive waiting-list control group in participating high schools. Data were collected using a self-administered questionnaire at two time-points over one school year: between November and December 2018 (baseline, pre-intervention) and between May and June 2019 (6-month follow-up, post-intervention). All participating students, including peer educators and peer recipients, completed the quantitative questionnaires at both time-points. These data were collected by trained members of the research team. Paper-based questionnaires were administered in classroom settings under the supervision of two members of the research team and a teacher. Members of the research team, professional health educators, high school staff, and students were blinded to group allocation (intervention versus control) only at baseline.

The primary outcome was the change in the prevalence of self-reported binge drinking between baseline and six-month follow-up among high school students. At both time points, participants were first provided with a standard drink chart listing the volumes of common alcoholic beverages corresponding to one unit of alcohol (36). Providing visual aids based on standard drink units has been shown to improve the accuracy and reliability of self-reported alcohol consumption among adolescents (37). Binge drinking in the past 30 days was then assessed using the following question: “How often in the past 30 days have you had five drinks or more on a single occasion?” This item is consistent with international definitions of binge drinking and has been widely used in adolescent populations (38). Participants answered this item using a five-point ordinal scale: “4 times or more” (coded 4), “3 times” (coded 3), “2 times” (coded 2), “1 time” (coded 1), and “I did not consume five or more drinks on a single occasion in the past 30 days” (coded 0). Given that the prevalence of binge drinking was the primary outcome of this study, responses were recoded into a binary variable to distinguish students who reported at least one binge drinking episode during the past 30 days (i.e., “4 times or more,” “3 times,” “2 times,” “1 time”) from those who reported none (i.e., “I did not consume five or more drinks on a single occasion in the past 30 days”). Additional analyses were also conducted using the original ordinal responses as a continuous outcome (coded from 0 to 4), thus reflecting binge drinking frequency during the past 30 days.

Further descriptive indicators of alcohol use were also collected at baseline. These included lifetime alcohol consumption (yes/no response) and the typical quantity of alcohol consumed on a drinking occasion. The latter measure was assessed using the following item: “How many alcoholic drinks do you consume on a typical drinking day?” (39), also presented after the standard drink chart (see above), with seven response options ranging from “I never drink alcohol” to “five or more standard drinks.”

Finally, socio-demographic variables collected at baseline included sex, age and family’s subjective financial situation. Regarding the latter measure, and in line with previous research (32), students were asked whether they perceived that their family could live comfortably (i.e., very comfortable, comfortable) or not (i.e., just managing, financial struggles at the end of some months, real financial hardships, very important financial hardships).

#### 2.3.3 Qualitative data collection

Qualitative data were collected at three different periods: (i) during the implementation of the SOFTPEERS program, logbooks were completed by professional health educators between January and March 2019, after each guidance session with peer educators; (ii) additional logbooks were filled in between March and May 2019, after each presentation of the prevention tools to peer recipients; and (iii) semi-structured interviews and focus groups were conducted between April and November 2019, following the implementation of the SOFTPEERS program. Qualitative data were collected by members of the research team trained in qualitative methods. Interviews and focus groups conducted with peer educators and peer recipients took place within the high schools, in dedicated rooms provided by high school staff. Interviews with the other participants were conducted either face-to-face or by phone. All sessions were audio-recorded with participants’ permission.

In the four high schools assigned to the intervention group, qualitative data were collected through 13 semi-structured interviews (average duration 41 min) and eight focus groups (average duration 47 min) conducted with various individuals involved in the SOFTPEERS program, including peer educators, peer recipients, school nurses, teachers, high school headmasters or vice headmasters, professional health educators, and members of the project team (see Table 1). In line with existing guidelines for evaluating key dimensions of process evaluation in complex interventions (25, 40), interview guides were developed to explore acceptability, implementation, and adaptation related to the SOFTPEERS program. All interview guides were structured around key thematic areas and included open-ended questions. While all guides shared a common core structure (e.g., reasons for engaging with the program, perceived facilitators and barriers, support received, perceived impact), they were also adapted to each type of participant involved in the program and covered the main themes relevant to their role. Specifically, peer educators were invited to reflect on their motivations to participate, the group dynamics, the process of designing and delivering their prevention action, and the perceived impact of the program. Teachers, as well as school nurses and high school headmasters or vice headmasters, were questioned about the delivery of the teacher-led session, their role in supporting the program, and their perception of student engagement. Professional health educators were invited to discuss the delivery and structure of the guidance sessions, their collaboration with peer educators and high school staff, and any adaptations made during implementation. Finally, project team members were asked about the coordination process, the implementation timeline, and the feasibility of deploying the intervention across multiple high schools.

**Table 1:** Overview of qualitative interviews conducted as part of the SOFTPEERS process evaluation.

Participants’ profiles	Number and type of interviews	Total number of interviewees
Peer educators	4 focus groups	10
Peer recipients	1 focus group	4
School nurses	6 individual interviews	6
Headmasters or vice headmasters	1 individual interview	1
Teachers	3 focus groups	7
Professional health educators	4 individual interviews	4
Members of the project team	2 individual interviews	2

In addition, logbooks were systematically completed by professional health educators to provide a complementary source of data related to implementation fidelity and contextual adaptations. A total of 28 logbooks were filled in after guidance sessions with peer educators, and nine after sessions in which peer-led prevention actions were delivered to peer recipients. These documents included both structured and open-ended items. Educators recorded basic session information (e.g., duration, number of participants), assessed the adequacy of session timing, and rated key aspects of group dynamics using a 5-point Likert scale (e.g., participation, atmosphere, richness of exchanges). In addition, they indicated whether the predefined objectives of the session were achieved and whether the proposed activity from the methodological guide had been followed. Each logbook also included space to describe the session in detail (e.g., session content, organization, materials used, debriefing), and to report contextual factors, obstacles encountered, or any adaptations made during implementation.

### 2.4 Data analysis

#### 2.4.1 Quantitative data analysis

Regarding quantitative data, statistical analyses were performed using SAS version 9.4. All tests were two-sided with an alpha level of 0.05. Data were analyzed according to the intention-to-treat principle. Descriptive statistics were first performed for binge drinking, lifetime alcohol consumption, and the typical quantity of alcohol consumed on a drinking occasion. Depending on the nature of the data, results are presented as means and standard deviations (SDs) or as medians or number and ranges (minimum–maximum) or percentage.

In order to evaluate whether the evolution of binge drinking over time differed between the two randomization groups, two mixed-effects models were conducted. The primary analysis focused on binge drinking prevalence, using mixed model for repeated measure. The secondary analysis examined binge drinking frequency, treated as a continuous score. In both models, fixed effects included randomization group (intervention vs. control), time (baseline, 6-month follow-up), and their interaction (group × time), which was used to estimate the intervention effect. Random intercepts were specified for individuals and high schools, and all models were adjusted for age, sex, family’s subjective financial situation, and typical quantity of alcohol consumed on a drinking occasion. Model estimates are presented with their 95% confidence intervals (CIs), with negative coefficients on the group x time interaction indicating a result in favor of the intervention group.

In addition to evaluating preliminary effectiveness and consistent with the objectives defined for this pilot study, the quantitative evaluation also included indicators of program demand assessed through both high school-level and student-level engagement with the SOFTPEERS intervention. At the high school level, demand was assessed through participation following initial agreement to take part in the study. At the student level, demand was operationalized via participation rates at baseline and at 6-month follow-up, as well as overall retention over time.

#### 2.4.2 Qualitative data analysis

A deductive thematic content analysis (41) was performed using NVivo version 12 (QSR International, Melbourne, Australia). All interviews and focus groups were transcribed verbatim and anonymized prior to analysis. The coding was carried out by an independent research team, distinct from those involved in data collection. The coding grid was developed *a priori* based on key dimensions of process evaluations for complex interventions (25), including acceptability (e.g., perceived usefulness, participant engagement), implementation (e.g., fidelity, dose, reach), and adaptations made during implementation (e.g., adjustments to local school constraints or participant needs). Inductive refinement was also allowed when new subthemes emerged from the data (41). Themes were analyzed separately for each participant group (e.g., peer educators, high school staff), and illustrative quotations were selected to reflect each theme.

Data from the logbooks were analyzed according to the type of information collected. Basic session characteristics (e.g., number of participants, session duration, achievement of predefined objectives, adherence to the proposed activity) were summarized using descriptive statistics (e.g., means, SDs, or proportions, depending on the nature of the data). Aspects of group dynamics among peer educators during guidance sessions, assessed using 5-point Likert scales (e.g., participation, group atmosphere, richness of exchanges), were also analyzed using summary descriptive statistics (e.g., means, SDs). Open-ended comments were reviewed to extract illustrative observations regarding contextual adaptations, obstacles encountered, and implementation-related issues (e.g., session content, materials used, contextual obstacles or constraints).

## 3 Results

The results are presented in accordance with the five key areas investigated in the present pilot study evaluating the SOFTPEERS intervention: demand, acceptability, implementation, adaptation, and preliminary effectiveness.

### 3.1 Demand: to what extent are high schools and students (i.e., peer educators and recipients) willing to engage with the SOFTPEERS program?

Of the nine high schools initially included in the pilot study, one in the control group subsequently declined to participate, resulting in eight participating high schools (*n* intervention = 4, *n* control = 4), enrolling a total of 2,704 students at baseline (see Figure 1). All eight high schools remained in the study and in the condition to which they had been allocated. At 6-month follow-up, 2,021 students completed the questionnaire, corresponding to an overall retention rate of 74.70% across intervention and control groups, while 683 students were lost to follow-up. The attrition rate in high schools from the intervention group (32.03%) was nearly twice as high as in those from the control group (16.63%). Further analysis indicated that this difference was primarily due to one high school in the intervention group where the 6-month follow-up data collection was severely disrupted by a local strike involving students, which made it particularly difficult to collect data at that time point. As a result, this high school had unusually high attrition (56.15%). When excluding this high school, attrition rates of students were closer between intervention and control groups (19.4% vs. 16.63%).

### 3.2 Acceptability: how is the SOFTPEERS program perceived in terms of relevance, appeal, and perceived value by students (i.e., peer educators and recipients) and program deliverers (i.e., professional health educators, project team members, and high school staff)?

Table 2 summarizes the main themes related to the evaluation of the acceptability of the SOFTPEERS program, along with illustrative excerpts from participant interviews. Peer educators considered the program appropriate and emphasized the importance of raising awareness among youth about the risks associated with alcohol consumption. Across all focus groups of peer educators interviewed, participants reported high levels of satisfaction with their involvement, describing the sessions as enjoyable and characterized by a friendly atmosphere. Some peer educators also described the experience as personally enriching, expressing positive memories of their participation and a willingness to take part in the program again. Similarly, members of the high school staff, such as vice headmasters and school nurses, appreciated the peer-to-peer approach. Peer recipients also expressed positive perceptions of the peer-to-peer approach, describing it as more open and pleasant than prevention led by adults, which some participants described as more moralizing. The proximity of peers was viewed as facilitating reflection on alcohol use and supporting self-regulation, notably by increasing awareness in drinking situations and knowledge about alcohol-related processes (e.g., metabolic elimination), as well as by enhancing perceived ability to regulate alcohol consumption. However, several peer recipients reported limited engagement with the peer-led actions, mainly due to time constraints within an already demanding school schedule. Others mentioned difficulties in identifying clear objectives or expectations regarding their attendance at peer-led actions. Project team members identified the program’s key strengths as its ability to address an important alcohol-related issue, convey relevant knowledge, and foster critical thinking. Teachers particularly appreciated the turnkey nature of their involvement, emphasizing that the 50-min session they led required only minimal preparation.

**Table 2:** Summary of main themes emerging from individual interviews and focus groups conducted as part of the SOFTPEERS process evaluation.

Process evaluation domains	Participants interviewed	Main themes	Representative quotations
Acceptability	Peer educators	Appropriate and relevant program (1/4 FG)	*“Me personally, it’s because today people our age go out to parties, drink like adults, and make all that normal”* (Peer educators, High school 1)
		Personally enriching experience (1/4 FG)	*“The advantage is that it enriches us, me, it really enriched me”* (Peer educators, High school 2, group 2)
	Vice headmasters and school nurses	Interest in the peer-to-peer approach (4/4 II)	*“The idea of being trained by peers, it’s interesting”* (Vice headmaster, High school 3) *“That’s what interested us, the peer-to-peer idea. Because we can really see it in the actions, when an adult does prevention in front of students, it’s not the same. We also saw that it was another method that was very appreciated by the students”* (School nurse, High school 1)
	Members of the project team	Relevance of addressing alcohol issues among adolescents (1/2 II)	*“The strong point is that it allows us to talk about the topic of alcohol over the long term and throughout the year, and to involve a group of young people in the high school who bring knowledge or at least some reflection on the topic”* (Member of the project team, interview 1)
	Teachers	Turnkey project (1/3 FG)	*“What was interesting is that it was a turnkey project. The presentation we had to do did not require any big preparation beforehand, a big preparation that would have required more work”* (Teachers, High school 4)
Implementation	School nurses	Heavy program to implement (3/3 II)	*“It was managing schedules, managing students, managing teachers, it was too heavy”* (School nurse, High School 2)
	Professional health educators	Logistical and scheduling difficulties (3/4 II)	*“There were two groups initially, so it was even more complicated because we had to find two available slots in the same week each time”* (Professional health educator, High school 1)
		Lack of time during sessions (4/4 II)	*“One hour in high school is 45 min. For example, there’s a schedule where you only get 45 min right at the beginning of the afternoon before the break. If we have that time slot, you only have 30 min”* (Professional health educator, High school 3)
Adaptation	Peer educators	Meetings outside school hours to complete the prevention action (2/4 FG)	*“We would meet outside”* (Peer educator, High school 2, group 2)

II, individual interview; FG, focus group.

### 3.3 Implementation: to what extent can the SOFTPEERS program be delivered as intended within the organizational and logistical constraints of real-world high school settings?

As shown in Table 2, several challenges related to the implementation of the SOFTPEERS program were reported by school nurses and professional health educators during the qualitative interviews. School nurses notably described the SOFTPEERS program as difficult to implement, highlighting the heavy coordination workload it entailed and the fact that it did not align well with students’ academic timetables. Professional health educators similarly reported significant logistical challenges, including the difficulty of scheduling sessions with peer educators and the limited time available to complete all the contents of the guidance sessions they supervised during the second phase of the program. These constraints sometimes prevented completion of the final activity involving relaxation exercises in session 6 to prepare peer educators to present their binge drinking prevention action to peer recipients. According to the logbooks completed by professional health educators, approximately 779 peer recipients were reached through the prevention actions delivered by peer educators. However, when supervising the organization of these activities, some professional health educators also reported issues with room availability and insufficient communication with teachers, which further complicated the implementation of prevention actions delivered by peer educators in some high schools.

### 3.4 Adaptation: to what extent was the SOFTPEERS program adapted to local constraints, practices and contexts of participating high schools?

Data related to program adaptations were collected through interviews conducted with peer educators (see Table 2), and through the logbooks completed by professional health educators. In response to time constraints encountered during the guidance sessions reported above, professional health educators occasionally shortened or adjusted the planned activities. In some high schools, an additional session was organized to allow peer educators to finalize the creation of their prevention tool. In addition, some peer educators took the initiative to meet outside of the scheduled sessions to continue working on their prevention projects.

### 3.5 Preliminary effectiveness: does the SOFTPEERS program show preliminary effectiveness in preventing binge drinking among students (i.e., peer educators and recipients) over a 6-month period?

**Figure 2:**
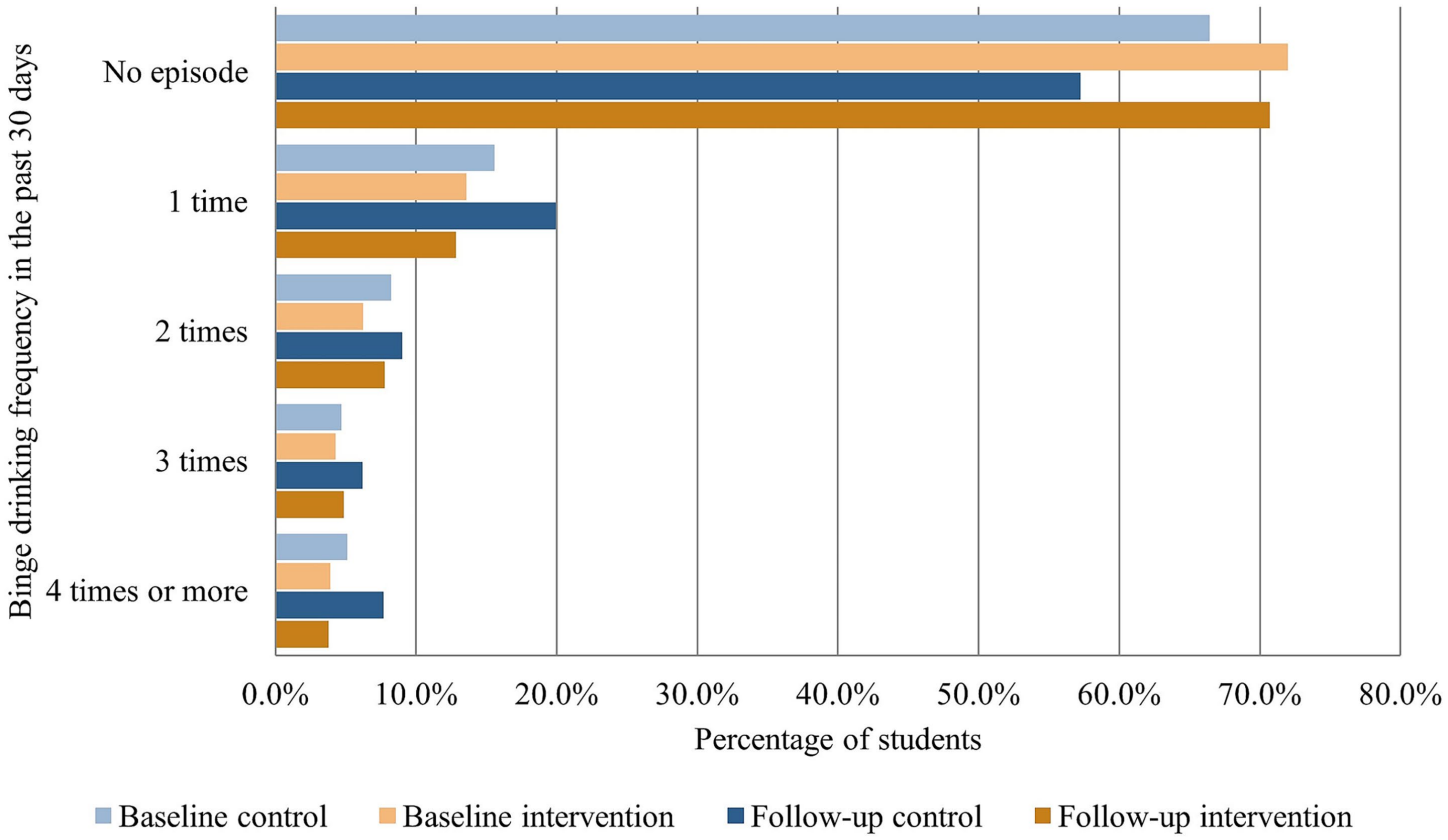
Distribution of binge drinking frequency in the past 30 days, by group and time point. For each group at baseline and at the 6-month follow-up, the bars represent the percentage of students in each category of binge drinking frequency in the past 30 days. Binge drinking frequency was assessed using the five-point ordinal item described in section 2.3.2 of the manuscript, coded from 0 (“I did not consume five or more drinks on a single occasion in the past 30 days”) to 4 (“4 times or more”). Within each group, percentages sum to 100% at each time point.

Eight high schools ultimately participated in the study (*n* intervention = 4, *n* control = 4), and 2,021 students (*n* intervention = 1,029; *n* control = 992) completed both baseline and 6-month follow-up questionnaires, and were thus included in the effectiveness analyses. Descriptive statistics for the whole sample and for intervention and control groups, including baseline differences on socio-demographic (e.g., grade level, age, sex, and family’s subjective financial situation, high school F-EDI score) and alcohol use variables (lifetime alcohol consumption, and the typical quantity of alcohol consumed on a drinking occasion), are presented in Table 3. The prevalence of binge drinking at baseline and 6-month follow-up is presented in Table 4, and the distribution of binge drinking frequency at both time points is illustrated in Figure 2. The mean age was 15.6 years (SD = 0.7), and 54.5% of participants were girls. At baseline, the prevalence of binge drinking in the past 30 days was 30.7%, with 14.5% of the sample reporting a single episode. In addition, 80.3% reported having consumed alcohol at least once in their life. Some differences were observed between the intervention and control groups on socio-demographic and alcohol drinking variables (e.g., age, lifetime alcohol consumption), including a lower prevalence of binge drinking in the past 30 days in the intervention group (28.0%) compared with the control group (33.6%) (*p* = 0.007). In contrast, the two groups did not differ significantly at baseline in terms of binge drinking frequency (*p* = 0.087).

**Table 3:** Sociodemographic characteristics and alcohol use at baseline.

Variables	Control group (*n* = 992)		Intervention group (*n* = 1,029)		Whole sample (*N* = 2021)		*p*-value
Grade level							<0.001
Grade 10	391	(39.4%)	595	(57.8%)	986	(48.8%)	
Grade 11	601	(60.6%)	434	(42.2%)	1,035	(51.2%)	
French version of the European Deprivation Index (F-EDI quintile)							<0.001
1–3 (low deprivation)	338	(34.1%)	275	(26.7%)	613	(30.3%)	
4–5 (high deprivation)	654	(65.9%)	754	(73.3%)	1,408	(69.7%)	
Sex							0.142
Male	468	(47.2%)	452	(43.9%)	920	(45.5%)	
Female	524	(52.8%)	577	(56.1%)	1,101	(54.5%)	
Age (years)							<0.001
Missing	1		1		2		
Mean (SD)	15.4 (0.6)		15.7 (0.7)		15.6 (0.7)		
Median (min; max)	15.0 (13; 18)		16.0 (12; 18)		16.0 (12; 18)		
Family’s subjective financial situation							0.887
Missing	13		16		29		
Can live comfortably	496	(50.7%)	510	(50.3%)	1,006	(50.5%)	
Cannot live comfortably	483	(49.3%)	503	(49.7%)	986	(49.5%)	
Lifetime alcohol consumption							<0.001
Missing	4		3		7		
No	134	(13.6%)	262	(25.5%)	396	(19.7%)	
Yes, at least once	854	(86.4%)	764	(74.5%)	1,618	(80.3%)	
Typical quantity of alcohol consumed on a drinking occasion							<0.001
Missing	17		11		28		
I never drink alcohol	246	(25.2%)	389	(38.2%)	635	(31.9%)	
Less than one standard drink	144	(14.8%)	110	(10.8%)	254	(12.7%)	
One standard drink	94	(9.6%)	81	(8.0%)	175	(8.8%)	
Two standard drinks	82	(8.4%)	96	(9.4%)	178	(8.9%)	
Three standard drinks	120	(12.3%)	82	(8.1%)	202	(10.1%)	
Four standard drinks	71	(7.3%)	74	(7.3%)	145	(7.3%)	
Five or more standard drinks	218	(22.4%)	186	(18.3%)	404	(20.3%)	

SD, standard deviation.

**Table 4:** Proportion of students reporting at least one binge drinking episode in the past 30 days, at baseline and 6-month follow-up.

	Control group (*n* = 992)		Intervention group (*n* = 1,029)	
Binge drinking episode	Baseline	6-month follow-up	Baseline	6-month follow-up
Missing	9	3	4	1
None	653 (66.4%)	566 (57.2%)	738 (72.0%)	727 (70.7%)
At least once	330 (33.6%)	423 (42.8%)	287 (28.0%)	301 (29.3%)

When comparing students with baseline and 6-month follow-up questionnaires included in the effectiveness analyses (*n* = 2,021) and students without 6-months follow-up data (*n* = 683), no significant differences were found in terms of sex, binge drinking prevalence, binge drinking frequency, or lifetime alcohol consumption at baseline (*p* > 0.05). However, students who completed both baseline and 6-month follow-up questionnaires were significantly older (*M* = 15.56, SD = 0.68 vs. *M* = 15.49, SD = 0.75; *p* = 0.002), were more likely to report never drinking alcohol on a typical drinking day at baseline (40.8% vs. 31.4%; *p* < 0.001), and were more likely to perceive that their family lives comfortably (49.8% vs. 44.5%; *p* = 0.03).

Regarding the effectiveness of SOFTPEERS on binge drinking prevalence (primary outcome), the time x group interaction indicated that the change in the prevalence of binge drinking in the past 30 days between baseline and 6-month follow-up was significantly smaller in the intervention group compared to the control group (*b* = −0.08, 95% CI [−0.12, −0.04]; see Table 5). More precisely, in the intervention group, the proportion of students reporting binge drinking increased from 28.0% at baseline to 29.3% at the 6-month follow-up (see Table 4), representing a 1.3 percentage point increase. In contrast, in the control group, this proportion increased from 33.6 to 42.8% (see Table 4), representing a 9.2 percentage point increase over the same period. At the 6-month follow-up, the prevalence of binge drinking was 13.5 percentage points higher in the control group than in the intervention group, resulting in a significant difference between the two groups at this time point (*p* < 0.001; see Table 4).

**Table 5:** Mixed models of intervention effects on binge drinking outcomes.

Outcomes	Estimate	Standard error	(95% CI)	*p*-value^a^
Proportion of students reporting at least one binge drinking episode in the past 30 days				
Intercept	−0.25	0.16	(−0.57, 0.07)	0.128
Group	0.003	0.02	(−0.03, 0.04)	0.871
Time	0.09	0.02	(0.06, 0.12)	<0.001
Time × group	−0.08	0.02	(−0.12, −0.04)	<0.001
Binge drinking frequency in the past 30 days				
Intercept	−0.05	0.42	(−0.88, 0.78)	0.898
Group	−0.002	0.04	(−0.09, 0.09)	0.961
Time	0.20	0.04	(0.13, 0.27)	<0.001
Time × group	−0.16	0.05	(−0.26, −0.06)	0.001

95% CI, 95% confidence interval.

a The analyses included fixed effects for group, time, and their interaction (group × time), with the control group at baseline as the reference. Models were adjusted for age, sex, family’s subjective financial situation, and typical quantity of alcohol consumed on a drinking occasion.

In relation to the frequency of binge drinking over the past 30 days (secondary outcome), the time × group interaction indicated that the change in binge drinking frequency between baseline and the 6-month follow-up was significantly smaller in the intervention group compared to the control group (*b* = −0.16, 95% CI [−0.26, −0.06]; *p* = 0.001; see Table 5). At the 6-month follow-up, the frequency of binge drinking was significantly lower in the intervention group compared to the control group (*p* < 0.001). This pattern is illustrated in Figure 2, which presents the distribution of binge drinking frequency in each group at both time points.

## 4 Discussion

The present pilot study aimed to assess the feasibility, acceptability, and preliminary effectiveness of the SOFTPEERS program, a peer-led intervention designed to prevent binge drinking among French high school students. In line with current recommendations (40), this study relied on the collection of both qualitative data to examine key aspects of the program’s implementation and quantitative data to assess its preliminary effectiveness. Quantitative findings from a pilot cluster randomized controlled trial involving 2,021 students suggest that SOFTPEERS has the potential to prevent the increase in binge drinking among French high school students. Over the 6-month follow-up period, the increase in the prevalence of binge drinking in the past 30 days was significantly smaller in the intervention group (+1.3 percentage points) compared to the control group (+9.2 percentage points), resulting in a between-group difference of 13.5 percentage points at the end of the study. This result was confirmed when considering binge drinking as a continuous outcome, with a significantly smaller increase in the number of binge drinking episodes in the past 30 days in the intervention group compared to the control group over the 6-month follow-up. While previous research has shown that peer-led interventions are effective in preventing alcohol use among adolescents (19), the present findings provide preliminary evidence that a peer-led approach may also help prevent a more specific pattern of alcohol consumption such as binge drinking. An important area for future research, however, is to more systematically investigate the mechanisms (i.e., mediators) underlying the effectiveness of peer-led interventions, in order to clarify how these interventions work, consolidate their effectiveness in preventing alcohol-related behaviors, and optimize their scalability among high school students (42, 43). For instance, a previous study has shown that the impact of a peer-led program on alcohol use was partly mediated by perceived descriptive norms (i.e., adolescents’ perceptions of how much their peers consume alcohol) (44). Descriptive norms have typically been associated with the reasoned pathway of health behavior change, notably through their influence on intentions (45). However, the observation that a peer-led intervention may also impact binge drinking, a behavior in which adolescents may engage even in the absence of intentions to do so (21), underscores the need to also examine mediators beyond the intentional pathway. In this regard, focusing on potential mediators involved in the social reaction pathway of behavior change may be particularly relevant (46), given the inherently social nature of peer-led interventions (14). For instance, future research could explore whether peer educators help their schoolmates refrain from engaging in binge drinking by influencing variables including behavioral willingness (i.e., reducing adolescents’ openness to engage in binge drinking during social events) and prototypes (i.e., challenging the positive image associated with peers who engage in binge drinking) (46).

Beyond preliminary effectiveness, the present study also explored the feasibility of the SOFTPEERS program through a qualitative process evaluation based on semi-structured individual interviews, focus groups, and logbooks completed by professional health educators after guidance sessions with peer educators. Overall, the findings indicated evidence of demand for the program among high schools and high levels of acceptability among the various individuals involved in the SOFTPEERS program (e.g., high school students, teachers). Of the nine high schools initially included, eight remained in the study, including all four schools allocated to the intervention group. After excluding one high school affected by local disruptions, the attrition rate in the intervention group was comparable to that observed in the control group. Such a result supports the overall feasibility of the study protocol and suggests that the SOFTPEERS program did not lead to any increased disengagement among students over 6 months (47, 48). Also, qualitative findings indicated high levels of satisfaction among peer educators, peer recipients, along with appreciation expressed by high school staff (vice headmasters and school nurses), teachers and members of the project team. The perceived relevance of targeting binge drinking and the peer-to-peer format appear to have contributed to the good acceptability of the program among the different actors involved. Among these findings, the receptiveness of high school students is particularly noteworthy, as they welcomed a peer-led intervention specifically targeting binge drinking despite the normalization of this behavior within youth culture (22), especially in France (5, 23). While adolescents may be prone to defensive reactions when participating in alcohol-related interventions, notably when these are mostly driven by high school staff or perceived as judgmental (18, 24), they may be more receptive to peer credibility, shared cultural references, and empathetic communication embedded in a peer-led approach (14). The high level of acceptability observed among high school students is particularly promising, as it represents a key condition for their adherence to peer-led interventions and for the successful implementation and broader dissemination of such programs targeting binge drinking in school settings (49, 50). Additionally, the program’s positive reception among teachers is particularly encouraging, as they were responsible for delivering the initial classroom session designed to stimulate early reflection on alcohol, introduce the intervention, and identify volunteer peer educators. While the supportive role of educational staff in peer-led interventions is increasingly recognized, there is still limited guidance on how this involvement can be structured to effectively support peer educators without undermining their central role (18). This role in the SOFTPEERS program was well received: teachers appreciated the turnkey nature of their involvement, the relevance of the topic, and the opportunity to stimulate students’ early reflection without the need for heavy preparation. Future research may more specifically explore how teachers perceive their potential roles in peer-led interventions in high school settings, examining for instance their expectations, perceived boundaries, and sense of legitimacy. These investigations could help further clarify how best to engage educational staff within a peer-led approach.

The qualitative data collected in this study also provided insights into the implementation and adaptation of the SOFTPEERS program during its delivery in high schools of the intervention group. Regarding implementation, several logistical and organizational challenges were reported by school nurses and professional health educators. The identified difficulties primarily reflected challenges in aligning the requirements of the SOFTPEERS program with the high school environment, including scheduling constraints and time limitations during the delivery of guidance sessions for peer educators, as well as coordination challenges in organizing peer-led binge drinking prevention actions for peer recipients. In response to these challenges, some adjustments were made, such as shortening certain planned activities during guidance sessions supervised by professional health educators, or organizing an additional session within the high school to give peer educators more time to complete their prevention action. Other adjustments could emerge from the peer educators themselves, who sometimes took the initiative to meet outside scheduled sessions to continue working on their prevention action. Taken as a whole, these adaptations can be interpreted as empirical adjustments to the constraints particularly encountered by peer educators and professional health educators during implementation. Such adjustments may reflect a context-sensitive process that helped sustain the delivery of the SOFTPEERS program, namely peer-led binge drinking prevention actions in intervention high schools (51). According to Stirman et al. (52), these adaptations observed in SOFTPEERS can be classified as content-level modifications, including “shortening/condensing” (e.g., adjusting the duration of certain activities during guidance sessions), “lengthening/extending” (e.g., organizing an additional guidance session in some high schools), and “adding elements” (e.g., peer educators meeting informally outside scheduled sessions to advance their prevention action). Rather than being considered as protocol deviations, these modifications illustrate both the capacity of individuals involved in SOFTPEERS to actively engage with its implementation, and the necessity of accounting for the real-world constraints they navigate when implementing the program (53). In line with current recommendations (25), some of the adaptations observed in this study could be integrated into the program in the future to facilitate its implementation, while preserving enough flexibility for local adjustment (54). This may include identifying specific activities within the guidance sessions that could be reduced if time constraints arise, and allowing the possibility to add an extra session when needed to support the finalization of the peer-led prevention action. In addition, efforts should be made to reduce the logistical burden placed on some of the professionals involved in the program (e.g., school nurses and professional health educators), in order to make the program easier to coordinate and deliver.

This pilot study presents several limitations that should be acknowledged. First, regarding quantitative data, the measure of binge drinking behavior in the past 30 days was assessed solely through self-report, which may have affected the validity of the results due to recall errors, social desirability bias and other reporting biases (55). Of note, when completing the measure of binge drinking, participants were first provided with a standard drink chart listing the volumes of common alcoholic beverages corresponding to one unit of alcohol (36), which has been shown to improve the accuracy and reliability of self-reported alcohol consumption among adolescents (37). However, future research should replicate the present results by combining self-reported data with biochemical measures of alcohol consumption, such as phosphatidylethanol, which has been shown to reliably quantify alcohol intake in adolescents (56). Second, members of the research team, professional health educators, high school staff, and students were blinded to group allocation (intervention versus control) only at baseline, which may have potentially biased present results (57). After baseline data collection, blinding could no longer be maintained, which may have influenced the behavior of high school students and high school staff. For instance, in the intervention group, awareness of group allocation may have affected students engagement with the SOFTPEERS program during peer-led prevention actions, as well as their responses to self-reported questionnaires at 6-month follow-up and to qualitative interviews (individual interviews and focus groups) conducted after the end of the program (58). Moreover, the absence of blinding after baseline may also have impacted the research team itself, including during questionnaire administration and qualitative data collection, potentially introducing subtle differences in how instructions were delivered or how interactions with participants unfolded (58). Nevertheless, all members of the research team were trained to follow a standardized protocol for both quantitative and qualitative data collection, which was implemented consistently across high schools to ensure the validity of the data collected. Third, although logbooks completed by professional health educators provided useful information on the overall implementation of the peer-led prevention actions in high schools of the intervention group, the present study did not include any standardized assessment of actual exposure at the individual level. As a result, it remains unclear which peer recipients were effectively exposed to the peer-led actions and to what extent. According to logbooks, approximately 779 students were reached, suggesting that a proportion of peer recipients in the intervention group may not have benefited from the peer-led prevention actions in a structured format (*n* intervention group = 1,029), although some of them may still have been indirectly influenced through more informal peer interactions, such as spontaneous conversations about the binge drinking prevention action implemented in the high school (18). Moreover, since some professional health educators reported implementation challenges in certain high schools (e.g., room availability or coordination issues), variability in exposure to peer-led prevention actions between or within high schools may also have influenced the observed impact. Future studies should incorporate specific measures of individual exposure to peer-led prevention actions among peer recipients, such as the number of minutes of exposure and the characteristics of the actions to which students were exposed (e.g., format, setting) (59, 60). These measures could help clarify how both the amount of exposure (e.g., time spent) and its modalities (e.g., passive vs. interactive formats, location) are related to intervention outcomes. In addition, it may be relevant to also explore informal or indirect exposure pathways, such as frequency of peer-to-peer conversations occurring outside the structured peer-led prevention actions (61). Taken as a whole, such investigations would help clarify the full range of exposure mechanisms at play in peer-led interventions and, in turn, inform future efforts to optimize both the structured dimension of the peer-led prevention actions within the SOFTPEERS program and the more informal pathways that may operate in high school settings (18). Fourth, while the qualitative process evaluation offered valuable insight into how the SOFTPEERS program was experienced and implemented, these findings were collected within a relatively bounded range of settings, as the participating high schools and their staff were all located within a single French educational authority (the Montpellier Academy). Since the interpretation of process evaluation findings is closely tied to the context in which they are produced (51), collecting qualitative data across a wider range of high schools would help consolidate the present findings and clarify how the program is experienced under varying conditions. In addition, most of the qualitative data, namely the individual interviews and focus groups, were gathered only after the intervention, which may limit insight into how experiences of the program evolved as it unfolded (51). Future research could therefore complement logbook data collected during implementation with additional interviews or focus groups conducted at several points throughout the program, rather than only retrospectively, to further consolidate and deepen the present findings.

## 5 Conclusion

The findings of this pilot study, based on a mixed-method approach, suggest that the SOFTPEERS program is a feasible, acceptable, and potentially effective intervention to prevent binge drinking among French high school students. Quantitative findings from a pilot cluster randomized controlled trial provide preliminary evidence that SOFTPEERS may help prevent the increase in the prevalence of binge drinking in this population. Demand for the program among high schools was supported by quantitative indicators of engagement, namely high school participation and student retention. Qualitative findings revealed good levels of acceptability among the individuals involved in the SOFTPEERS program, including peer educators. Despite some logistical and organizational challenges during implementation of the program, several adjustments were made, particularly by professional health educators and peer educators, which helped sustain both the preparation of peer educators through guidance sessions and the delivery of peer-led prevention actions in the high schools.

This pilot study represents an initial step in the evaluation of the SOFTPEERS program, with several important perspectives for future research. In line with current recommendations on the evaluation of complex interventions (62), an important next step will be to conduct a full-scale cluster randomized controlled trial of SOFTPEERS to confirm the preliminary evidence of effectiveness observed in this pilot study regarding adolescents’ binge drinking, and to test the program on a larger population across more diverse settings (e.g., urban and rural high schools, with a greater number of high schools represented across the quintiles of the F-EDI score). Such a future trial should also include an embedded process evaluation to examine the mechanisms underlying the intervention’s effects (e.g., role model of peer educators), the organizational factors shaping implementation (e.g., coordination challenges in organizing peer-led binge drinking prevention actions), as well as how these dimensions interact with broader contextual conditions (e.g., high school size) to inform transferability of the SOFTPEERS program (63). Beyond this perspective, future research could also explore the impact of SOFTPEERS through longer follow-up periods, such as across several school years (32), investigate the sustainability of effects after the intervention has ended (64), and evaluate the cost-effectiveness of SOFTPEERS (65).

## Data Availability

The raw data supporting the conclusions of this article will be made available by the authors, without undue reservation.
